# Asthma-Related ED Visits Among Children in the United States: A Cross-Sectional Analysis of Associated Factors Using the National Hospital Ambulatory Medical Care Survey (NHAMCS), 2006-2020

**DOI:** 10.7759/cureus.88970

**Published:** 2025-07-29

**Authors:** Zainab T O. Omar, Somtochi Udekwe, Anthonia N Njoku, Oluchi Solomon-Anucha, Ibiwumi O Atolagbe, Olamma A Dike, Chinasa Okeke-Chikelu, Mofiyinfoluwa Fagbemi, Okelue E Okobi

**Affiliations:** 1 Pediatrics, St. Joseph's Regional Medical Center, Paterson, USA; 2 Diabetes and Endocrinology, California Institute of Behavioral Neurosciences and Psychology, Fairfield, USA; 3 Spine Clinic, University of Texas (UT) Southwestern Medical Center, Frisco, USA; 4 Family Medicine, Abia State University, Abia, NGA; 5 Medicine, University of Ibadan, Ibadan, NGA; 6 Family Medicine, Abia State University Teaching Hospital, Altona, CAN; 7 Medicine, Windsor University School of Medicine, Cayon, KNA; 8 Family Medicine, All Saints University School of Medicine, Roseau, DMA; 9 Family Medicine, Larkin Community Hospital Palm Springs Campus, Hialeah, USA; 10 Family Medicine, IMG Research Academy and Consulting, Homestead, USA

**Keywords:** emergency department, health disparities, nhamcs, pediatric asthma, race and ethnicity, socioeconomic factors

## Abstract

Background

Asthma remains a leading cause of ED visits among children in the United States. Despite clinical advancements, disparities in asthma care and control persist, particularly among socioeconomically and racially marginalized populations.

Objective

This study examines trends and sociodemographic factors associated with asthma-related ED visits in children using data from the National Hospital Ambulatory Medical Care Survey (NHAMCS) from 2006 to 2020.

Method

To achieve the study objective, we analyzed pediatric ED visit data (ages 0-17) from NHAMCS (2006-2020), applying survey weights to account for the complex sampling design. Descriptive statistics compared children with and without asthma-related ED visits. Logistic regression identified associations between asthma-related visits and demographic variables, including age, sex, race/ethnicity, and socioeconomic factors (ambulance arrival and insurance type).

Results

The findings indicated that asthma-related visits showed no significant decline over the study period. Medicaid insurance was associated with higher odds of asthma-related visits, while uninsured children were underrepresented. Non-Hispanic Black children (OR = 5.36; 95% CI: 1.59-18.02) and non-Hispanic “Other” children (OR = 54.93; 95% CI: 10.87-277.63) had significantly higher odds compared to non-Hispanic Whites. Gender disparities favored males in asthma-related visits, and ambulance use did not differ significantly between groups.

Conclusion

Asthma-related ED visits in children remain prevalent and unequally distributed across racial and socioeconomic lines. These disparities emphasize the need for targeted, equity-driven interventions to strengthen outpatient asthma management and reduce reliance on emergency care.

## Introduction

Asthma remains one of the most common chronic illnesses in children in the United States, posing a major public health issue due to its effects on quality of life, healthcare utilization, and long-term outcomes [1]. According to the United States CDC, epidemiologists and clinical researchers concur that the burden of asthma is higher among children compared to adults [2]. Despite the availability of effective management strategies and medications, asthma continues to be a leading cause of ED visits, hospitalizations, and missed school days among children [3, 4]. The long-term persistence of asthma-related ED visits appears to indicate a sustained lack of asthma control and preventive care, as well as a pattern of structural and systemic disparities in healthcare access and social determinants of health [5].

The socioeconomic conditions under which a child resides are key factors in how they are exposed to asthma triggers, whether they can receive continuous care, and how well they can manage the disease overall [6]. This has been supported by various studies showing that children living in economically deprived groups are disproportionately exposed to uncontrolled asthma and acute exacerbations necessitating emergency treatment [7, 8]. Issues such as poverty, poor-quality housing, limited access to primary healthcare professionals, caregivers' education levels, food insecurity, and insurance coverage are all significant barriers to effective asthma management and subsequent care [9, 10]. Moreover, for marginalized communities and those living in more polluted and less green environments, symptoms may be further exacerbated [11].

Data available from EDs, especially those representing the national population, such as the National Hospital Ambulatory Medical Care Survey (NHAMCS), are highly informative regarding trends and disparities in healthcare use [12, 13]. NHAMCS provides comprehensive data on the number of patients visiting outpatient and EDs of hospitals nationwide, making it a crucial tool for public health surveillance and policymaking [14]. The NHAMCS data from 2006 to 2020 offer researchers the opportunity to analyze trends in asthma-related ED visits among children over time and to explore their association with factors such as income level, race, and insurance status [15, 16].

Understanding these associations is essential for identifying vulnerable groups, implementing interventions, and designing policy programs aimed at addressing health disparities. For instance, children in low-income or minority groups consistently bear a disproportionately high burden of asthma-related ED visits [17]. Therefore, public health officials and policymakers may need to allocate more resources toward school-based asthma education programs, environmental regulation measures, or community health worker outreach in affected communities [18]. Furthermore, identifying patterns in asthma-related ED visits, such as increases during periods of economic recession, policy changes, or environmental crises, can support trend analysis and improve preparedness initiatives [19].

Despite significant advancements in asthma research and treatment over recent decades, persistent racial and socioeconomic disparities in emergency pediatric asthma care underscore the need for further investigation [20]. This study aims to examine the association between socioeconomic factors and asthma-related emergency department visits among children in the United States from 2006 to 2020, using data from the National Hospital Ambulatory Medical Care Survey (NHAMCS) [21].

## Materials and methods

Study design and data source

This study employed a retrospective, cross-sectional design using data from the National Hospital Ambulatory Medical Care Survey (NHAMCS) for the years 2006 to 2020 [21]. NHAMCS is a nationally representative survey conducted annually by the National Center for Health Statistics (NCHS), part of the CDC. It collects detailed information on patient visits to EDs in non-institutional general and short-stay hospitals across the United States, excluding federal, military, and Veterans Affairs hospitals. The survey uses a complex, multistage probability sampling design, enabling national-level estimates of ED utilization.

Study population

The analysis was limited to children aged 0-17 years who visited an ED between 2006 and 2020. Adult patients were excluded. The final unweighted sample included 42,526 pediatric ED visits. After applying NHAMCS sampling weights to generate national estimates, this corresponded to approximately 10,399 weighted visits, as recommended for statistical analysis.

Primary outcome

The main outcome variable was whether the ED visit was asthma-related, defined as the presence of an International Classification of Diseases (ICD-9-CM) diagnosis code for asthma (code 493) in any of the top three physician-assigned diagnosis fields (diag1-diag3) recorded for each ED visit in NHAMCS. A binary variable (visit_asthma) was generated to indicate asthma-related ED visits (1 = asthma-related, 0 = non-asthma).

Independent variables

The study considered a range of sociodemographic and clinical variables as independent predictors of asthma-related ED visits. Age was included as a continuous variable, measured in completed years. Gender was coded as male or female based on the recorded biological sex of the child at the time of the visit. Race and ethnicity were harmonized across all survey years using either the RACEETH or RACERETH variables, depending on availability, and categorized into four mutually exclusive groups: Non-Hispanic White, Non-Hispanic Black, Hispanic, and Non-Hispanic Other. Insurance status, representing the primary expected source of payment for the ED visit, was derived from a combination of variables (PAYPRIV, PAYMCAID, PAYMCARE, PAYOTH) and standardized across survey years. It was categorized into three groups: Medicaid, Private Insurance, and Other/Uninsured. The mode of arrival at the ED was also included, specifically, whether the child arrived by ambulance. This was coded as a binary variable (Yes/No), using ARRIVE for years 2006-2008 and ARREMS for 2009 onward, to maintain consistency across the dataset. Finally, the survey year was included both as a continuous covariate and, in some analyses, as a categorical variable to assess trends over time in asthma-related ED utilization.

Data processing

Data for each year (2006-2020) were imported, cleaned, and standardized individually in Stata 18. Key variables were harmonized across survey years (e.g., race/ethnicity, insurance types, ambulance arrival) to ensure consistency. The files were then appended, and a final analytic dataset was generated, restricted to pediatric visits only, as shown in Table 1.

**Table 1:** Cleaned first 10 rows of the final analytic dataset after preprocessing (NHAMCS pediatric ED visits, 2020 sample). This table displays a sample of the first 10 rows from the cleaned dataset used in the analysis, limited to pediatric ED visits recorded in 2020 from the NHAMCS database. year: Survey year (all entries shown are from 2020); edwt: ED visit weight used to generate nationally representative estimates (not shown due to masking); cpsum: Clustered primary sampling unit (masked for confidentiality); cstratm: Clustered stratification variable (masked for confidentiality); gender: Sex of the patient (male or female); race/ethnicity: Patient’s race/ethnicity, categorized into major NHAMCS-defined groups; age (in years): Child's age in completed years at the time of the ED visit; visit_asthma: Binary indicator for whether the ED visit was asthma-related (1 = yes; 0 = no); insurance type: Expected primary source of payment for the ED visit (e.g., Medicaid, private); arrival by ambulance: Binary indicator for mode of arrival (1 = yes, arrived by ambulance; 0 = no, arrived by other means). NHAMCS: National Hospital Ambulatory Medical Care Survey.

Year	Gender	Race/Ethnicity	Age (in years)	visit_asthma	Insurance Type	Arrival by Ambulance
2020	Male	Non-Hispanic Black	8	0	Private	1
2020	Female	Hispanic	15	1	Medicaid	1
2020	Male	Non-Hispanic Black	6	0	Medicaid	0
2020	Female	Non-Hispanic Black	0	0	Medicaid	0
2020	Female	Hispanic	4	1	Medicaid	1
2020	Female	Non-Hispanic Black	10	0	Private	0
2020	Male	Hispanic	6	0	Medicaid	
2020	Male	Non-Hispanic Black	10	0	Medicaid	0
2020	Female	Non-Hispanic Black	10	0	Medicaid	1
2020	Female	Non-Hispanic Black	13	0	Medicaid	0

Statistical analysis

Descriptive statistics summarized the characteristics of ED visits stratified by asthma status. Design-based F-tests (for categorical variables) and t-tests (for continuous variables) were used instead of standard chi-square and t-tests, respectively, accounting for the NHAMCS complex survey design (stratification, clustering, and weights) to ensure valid national estimates and accurate statistical inference. A survey-weighted logistic regression model was used to estimate the adjusted odds of asthma-related ED visits associated with sociodemographic factors, reporting ORs with 95% CIs and p-values. In addition, two figures were generated to visually support the findings of this study. The first was a line graph depicting the temporal trend in asthma-related ED visits among children from 2006 to 2020, offering insight into patterns over time. The second was a forest plot that illustrated the adjusted odds ratios derived from the multivariable logistic regression model. All analyses were performed using Stata 18.0 (StataCorp LLC, College Station, TX). A significance level of p < 0.05 was used for all statistical tests.

Ethical consideration

The NHAMCS dataset is publicly available and fully de-identified; hence, this study was exempt from institutional review board (IRB) approval. No identifiable patient data were accessed or used. The study was conducted in accordance with the principles outlined in the Declaration of Helsinki.

## Results

Table 2 below indicates the weighted demographic and clinical attributes of pediatric ED visits by asthma diagnosis status. 

**Table 2:** Weighted demographic and clinical characteristics of pediatric ED visits by asthma diagnosis status, NHAMCS 2006-2020 (Unweighted n = 42,526; Weighted N = 10,399) This table compares characteristics of children presenting to the ED for asthma-related visits versus non-asthma-related visits. Weighted percentages and means reflect national estimates. Statistical comparisons include design-based F-tests for categorical variables and t-tests for continuous variables, accounting for the complex survey design of NHAMCS. Asterisks indicate statistical significance: p < 0.05, p < 0.01, *p < 0.001 –: Intentionally left blank; N: Number of patients; NHAMCS: National Hospital Ambulatory Medical Care Survey.

Variable	Asthma-Related ED Visit (N = 327)	Non-Asthma Visit (N = 10,072)	T-test	F-test	p-value
Age (in years)	7.04 ± 3.39	7.01 ± 5.85	-0.03	-	0.978
Gender	-	-	-	4.09	0.04*
Male	267 (82%)	5,438 (54%)	-	-	-
Female	60 (18%)	4,634 (46%)	-	-	-
Arrival by Ambulance	-	-	-	0.001	0.973
Yes	14 (4%)	389 (4%)	-	-	-
No	313 (96%)	9,013 (96%)	-	-	-
Insurance	-	-	-	3.14	0.06
Medicaid	243 (74%)	4,933 (49%)	-	-	-
Private	79 (24%)	3,356 (33%)	-	-	-
Other/Uninsured	5 (2%)	1,784 (18%)	-	-	-
Race/Ethnicity	-	-	-	15.12	p < 0.001***
Non-Hispanic White	79 (24%)	6,302 (63%)	-	-	-
Non-Hispanic Black	126 (39%)	1,944 (19%)	-	-	-
Hispanic	20 (6%)	1,653 (16%)	-	-	-
Non-Hispanic Other	102 (31%)	174 (2%)	-	-	-

The mean age of children presenting with asthma-related ED visits was 7.04 years (±3.39), nearly identical to those with non-asthma-related visits (7.01 years ±5.85), and this difference was not statistically significant (p = 0.978), suggesting that age was not a distinguishing factor between the two groups. In terms of gender, a statistically significant difference was observed (F = 4.09, p = 0.04). Males accounted for a markedly higher proportion of asthma-related ED visits, representing 267 (82%) of the cases, compared to females at 60 (18%). Among non-asthma visits, the gender distribution was more balanced, with 5,438 (54%) males and 4,634 (46%) females. The observed male predominance in asthma-related ED visits may reflect both biological susceptibility, such as smaller airway diameter in boys, and differential environmental exposures or healthcare-seeking behaviors that influence asthma control and exacerbation risk during childhood.

Arrival mode did not significantly differ between groups, with ambulance use at only 14 (4%) in both asthma and non-asthma visits (F = 0.001, p = 0.973). This suggests that asthma-related visits were not more likely to be perceived as emergencies requiring ambulance transport, possibly reflecting similar levels of acute severity or caregiver behavior across groups. Regarding insurance status, a marginally significant association was identified (F = 3.14, p = 0.06). Children with asthma-related ED visits were more likely to be covered by Medicaid, 243 (74%) compared to 4,932 (49%) of non-asthma visits. Conversely, private insurance was less common among asthma visits, 79 (24%) vs. 3,356 (33%), and “Other/Uninsured” coverage was notably lower among asthma cases, 5 (2%) vs. 1,784 (18%), underscoring the role of socioeconomic disadvantage in asthma-related ED utilization.

Striking racial and ethnic disparities were also evident (F = 15.12, p < 0.001). Non-Hispanic White children comprised just 79 (24%) of asthma-related visits, compared to 6,301 (63%) of non-asthma visits. In contrast, Non-Hispanic Black children represented 126 (39%) of asthma visits, more than double their share in the non-asthma group (1,944; 19%). Additionally, non-Hispanic children identified as “Other” racial groups accounted for 102 (31%) of asthma visits but only 173 (2%) of non-asthma visits. These findings highlight the disproportionate burden of asthma-related ED visits among racial and ethnic minority children, reflecting persistent disparities in pediatric asthma management, environmental exposures, and access to routine care.

**Figure 1:**
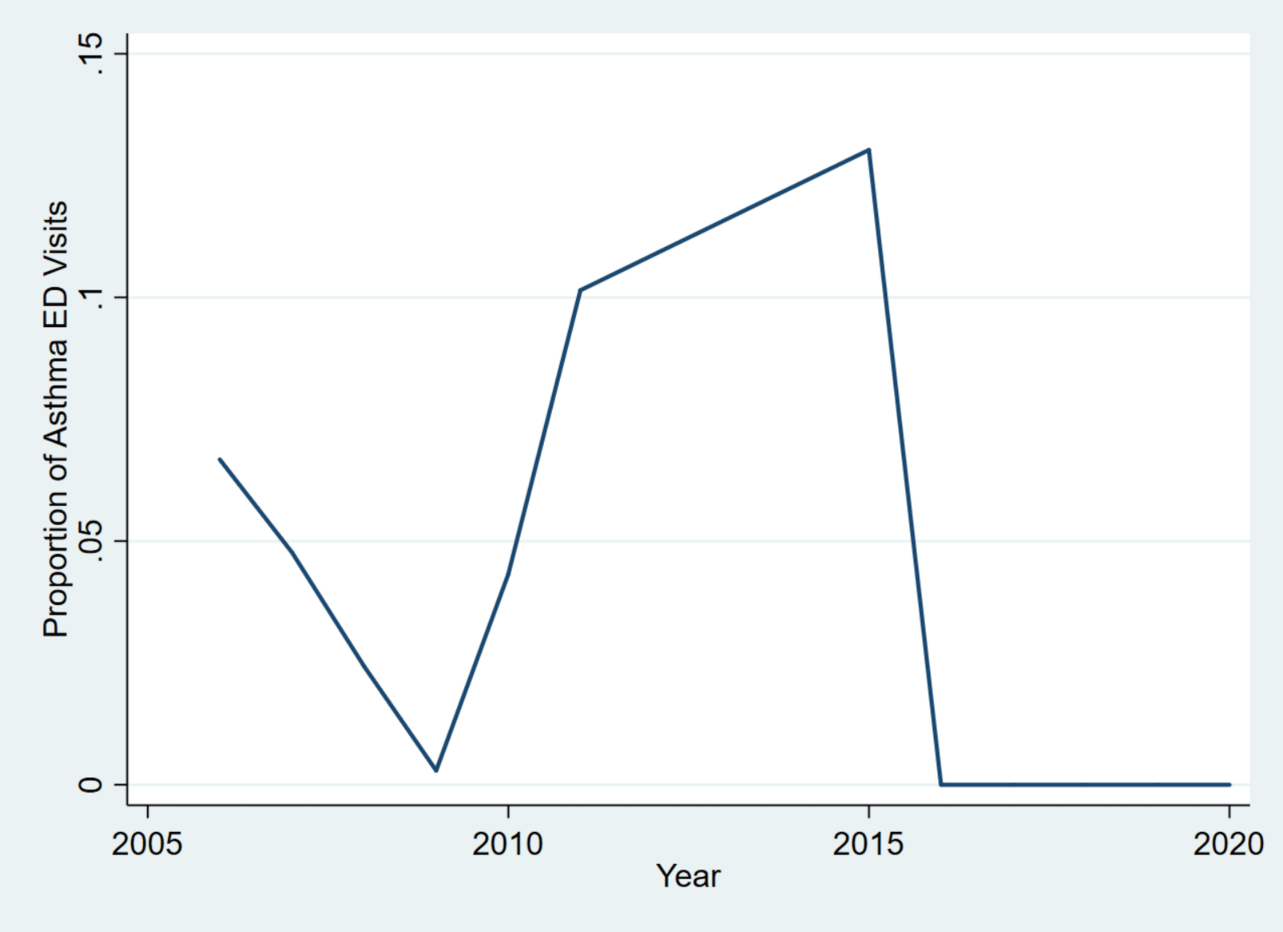
Trend in asthma-related ED visits among children in the United States, 2006-2020. This figure displays the annual proportion of ED visits attributed to asthma among children aged 0-17 years in the United States, using weighted data from the National Hospital Ambulatory Medical Care Survey (NHAMCS) from 2006 to 2020. The trend fluctuates between 2006 and 2015, with notable peaks in 2011 (10.1%) and 2015 (13.0%), followed by a dramatic decline to 0% from 2016 through 2020. The sharp drop, especially in 2020, may reflect the impact of the COVID-19 pandemic on ED utilization patterns, changes in care-seeking behavior, and potential improvements in asthma control due to reduced exposures to the virus and environmental triggers.

To visualize the temporal dynamics of asthma-related ED visits among children, a trend analysis was conducted using weighted annual proportions from 2006 to 2020. The resulting line graph, as indicated by Figure 1, illustrates fluctuations in visit rates over time and highlights key shifts potentially influenced by public health events.

The trend indicates no consistent long-term decline in pediatric asthma-related ED visits up to 2015, highlighting persistent gaps in asthma management and prevention. However, the absence of cases from 2016 to 2020 suggests either possible data or reporting limitations, or more likely, an external influence such as the COVID-19 pandemic that significantly altered healthcare utilization and may have temporarily improved asthma outcomes through reduced exposure to common asthma triggers.

Multivariable analysis of factors associated with asthma-related ED visits

Table 3 presents results from the survey-weighted logistic regression model assessing the association between sociodemographic factors and the likelihood of an asthma-related ED visit among children.

**Table 3:** Survey-weighted multivariable logistic regression assessing factors associated with asthma-related ED visits in children, NHAMCS 2006-2020. This table presents adjusted ORs and 95% CIs from a survey-weighted logistic regression model. The outcome variable is whether the ED visit was for asthma. Independent variables include age, sex, mode of arrival, insurance type, and race/ethnicity. The reference groups are: Female (sex), Medicaid (insurance), and Non-Hispanic White (race/ethnicity). ORs greater than 1 indicate higher odds of asthma-related visits relative to the reference group. Asterisks indicate statistical significance: p < 0.05, p < 0.01, *p < 0.001 –: Intentionally left blank; NHAMCS: National Hospital Ambulatory Medical Care Survey.

Visit was for asthma diagnosis	OR	95% CI	p-value
Age (in years)	1.07	1.01-1.15	0.03*
Gender	-	-	-
Male	3.76	0.89-15.90	0.07
Ambulance arrival	-	-	-
Yes	1.58	0.18-13.59	0.68
Insurance	-	-	-
Private	0.73	0.25-2.12	0.57
Other/Uninsured	0.03	0.00-0.40	0.01*
Race/Ethnicity	-	-	-
Non-Hispanic Black	5.36	1.59-18.02	0.01*
Hispanic	0.96	0.15-6.12	0.97
Non-Hispanic Other	54.93	10.87-277.63	<0.001***

Age was found to be a statistically significant predictor of asthma-related ED visits. With each one-year increase in age, the odds of an asthma-related ED visit increased by 7% (OR = 1.07, 95% CI: 1.01-1.15; p = 0.03). This finding suggests that older children within the sampled pediatric age range were modestly more likely to present to the ED for asthma, potentially reflecting delayed onset, cumulative exposure, or underdiagnosis in earlier years.

Gender differences were not statistically significant, although male children showed higher odds of an asthma-related visit compared to females (OR = 3.76, 95% CI: 0.89-15.90; p = 0.07). While this result approached significance, the wide confidence interval indicates variability and potential lack of power.

Arrival by ambulance was not significantly associated with asthma-related visits (OR = 1.58, 95% CI: 0.18-13.59; p = 0.68). This suggests that the mode of arrival, often considered a proxy for clinical severity, did not differ meaningfully between asthma and non-asthma ED visits after adjusting for covariates.

Regarding insurance status, children with “Other/Uninsured” coverage had significantly lower odds of asthma-related ED visits compared to those covered by Medicaid (OR = 0.03, 95% CI: 0.00-0.40; p = 0.01). This could reflect access disparities, differential diagnosis coding, or utilization behavior. Children with private insurance also had lower odds (OR = 0.73, 95% CI: 0.25-2.12), though the association was not statistically significant (p = 0.57).

**Figure 2:**
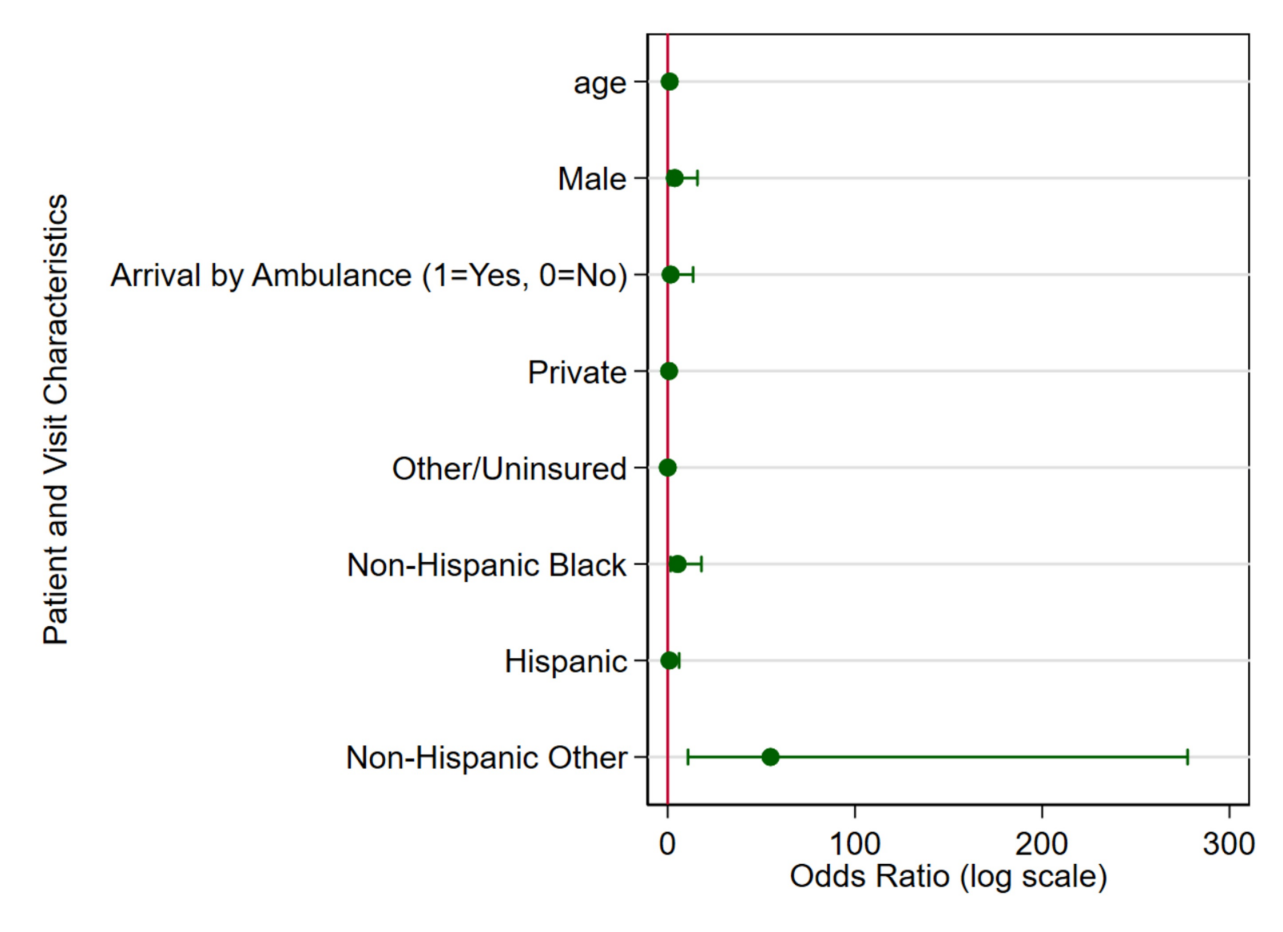
Forest plot of adjusted ORs and 95% CI for factors associated with asthma-related ED visits among children, NHAMCS 2006-2020. This plot presents the adjusted ORs from a survey-weighted logistic regression model evaluating the association between demographic and clinical factors and the likelihood of an ED visit being related to asthma. Each point represents the OR for a given variable, with horizontal lines indicating 95% CIs. The vertical reference line at OR = 1 denotes no association. Values to the right of the line indicate increased odds of an asthma-related visit, while those to the left indicate decreased odds. Significant associations were observed for age, race/ethnicity, and insurance status. NHAMCS: National Hospital Ambulatory Medical Care Survey.

Striking disparities emerged in race/ethnicity. Compared to Non-Hispanic White children, Non-Hispanic Black children had significantly higher odds of asthma-related ED visits (OR = 5.36, 95% CI: 1.59-18.02; p = 0.01), highlighting a substantial disparity. The odds were most elevated among children identified as Non-Hispanic Other (OR = 54.93, 95% CI: 10.87-277.63; p < 0.001), suggesting a profound overrepresentation of this group in asthma ED visits. This may reflect a combination of social, environmental, and access-related factors that disproportionately affect children in this category. In contrast, Hispanic children did not differ significantly from Non-Hispanic Whites in terms of asthma-related ED visit odds (OR = 0.96, 95% CI: 0.15-6.12; p = 0.97). Figure 2 below shows the forest plot of adjusted odds ratios (ORs) and 95% confidence intervals for factors associated with asthma-related ED visits among children.

## Discussion

This study analyzed national ED data from the NHAMCS from 2006 to 2020 to evaluate the burden of asthma-related visits among children and examine associated demographic and socioeconomic factors. Using survey-weighted methods, we observed key disparities in asthma-related ED use, particularly related to race/ethnicity, insurance coverage, and socioeconomic vulnerability. Our findings indicate that the overall proportion of ED visits due to asthma remained relatively stable over the 15 years, suggesting limited progress in reducing acute asthma exacerbations requiring emergency care. This may reflect persistent challenges in asthma control, access to preventive care, or environmental risk factors.

The observed sharp decline in asthma-linked pediatric ED visits, especially in 2020, might reflect either an actual reduction in exacerbations or pandemic-period disruptions in healthcare coding and utilization. Thus, the exceptional 2020 decrement aligns with broader trends of avoided ED care due to COVID-19 fears, significant shifts to telehealth for mild to moderate cases, and potential underreporting in the NHAMCS data. Nevertheless, one cannot rule out actual improvements in asthma control, given that reductions in viral exposures (as a result of lockdowns) and decreased air pollution might have significantly reduced asthma triggers. Notably, public health measures during the COVID-19 pandemic, such as masking, school closures, and social distancing, led to reduced circulation of common respiratory viruses, particularly rhinovirus, a known trigger of pediatric asthma [22, 23]. This likely contributed to the decreased ED utilization, as supported by recent findings [23]. Moreover, even as pre-pandemic studies disclosed stable or increasing asthma ED visits [6, 11-14], our findings regarding the extreme decline contrast with more modest reductions in other notable databases, indicating potential data limitations. Prospective studies should therefore cross-validate NHAMCS with claims data to determine whether the observed trend reflects real declines or methodological artifacts.

Our results also show that age was not a significant distinguishing factor, with mean ages nearly identical between asthma and non-asthma visits. However, children with asthma-related visits were more likely to be male, diverging from some earlier studies that showed higher asthma prevalence in young females [1-6, 13-18]. This finding also contradicts previous research, including national surveillance data, which has shown either a higher asthma prevalence among females or a more even distribution, particularly among adolescents and adults [1-6, 13-18, 22]. Notably, the National Center for Health Statistics reported that females aged 0-17 years had a higher asthma prevalence than males during 2008-2010 (9.2% vs. 7.0%) [22]. This gender shift may reflect age-related patterns, hormonal influences during adolescence, or differences in care-seeking behavior.

A notable racial and ethnic disparity emerged from both descriptive and multivariable analyses. Non-Hispanic Black children had over five times greater odds of presenting with asthma-related ED visits compared to Non-Hispanic White children. Similarly, children categorized as Non-Hispanic "Other" (which may include multiracial, Asian, and Native American) had dramatically elevated odds, with an adjusted odds ratio of 54.9. These findings emphasize longstanding concerns about inequities in asthma prevalence, severity, and access to preventive care among minority populations.

Insurance status was also a significant indicator of ED utilization. A majority, 243 (74%), of asthma visits were among children with Medicaid, while privately insured and uninsured/other categories were less represented. The logistic model further confirmed that uninsured/other children were significantly less likely to visit the ED for asthma, possibly due to underutilization or barriers to care access. This finding may reflect underdiagnosis or delayed care among uninsured children, which can worsen outcomes. In contrast, arrival by ambulance was not significantly associated with asthma-related visits. This suggests that most pediatric asthma visits, while urgent, may not always present with life-threatening severity or may reflect caregiver-perceived urgency rather than objective clinical need.

Despite advances in asthma management, the findings of this study reinforce what many clinicians and public health experts already know: racial and socioeconomic disparities in pediatric asthma care remain deeply rooted. The finding that Non-Hispanic Black and "Other" children had significantly higher odds of asthma-related ED visits reflects longstanding inequities in healthcare access and environmental exposures [8, 18]. Children from marginalized communities often live in areas with poor air quality, inadequate housing, and limited access to primary care or asthma education [5, 11]. These factors make it harder to attain asthma control and increase the likelihood of emergency visits. Similarly, the overrepresentation of children with Medicaid in asthma-related ED visits highlights the barriers faced by lower-income families in accessing consistent outpatient care and preventive services [17, 10]. These are not just clinical challenges; they are systemic and structural, and they require a broader, more inclusive approach to health equity.

To effectively reduce asthma-related ED visits in the future, proactive and community-based interventions should be considered. Moreover, there is a need for targeted and equitable public health efforts to reduce asthma-related health burdens. Programs that bring asthma education to schools, train community health workers, and offer caregiver support have been found to improve asthma care outcomes while also reducing incidences of acute asthma episodes [2, 19]. Furthermore, earlier studies have shown that significant reductions in ED visits can be achieved when families understand how to manage asthma, especially in high-risk environments [7, 9]. Data from large-scale surveys like NHAMCS are valuable tools for identifying the most affected populations and informing where more resources should be allocated [12, 13]. To address the above gaps, policies are needed to improve housing, increase access to care, and support families with asthma patients long before the condition becomes an emergency.

Limitations

Despite the strengths of using a nationally representative dataset spanning 15 years, this study has several limitations. First, the cross-sectional design of the NHAMCS limits the ability to infer causal relationships between the identified predictors and asthma-related ED visits. Second, the diagnosis of asthma was based on provider-reported codes and not validated against clinical records, which may introduce misclassification bias. Third, the NHAMCS data do not include detailed clinical information such as asthma severity, medication use, or comorbidities, factors that are important in understanding ED utilization. Missing data and small sample sizes in certain subgroups, especially among asthma cases, may have reduced statistical power and precision in estimates. Lastly, the dataset does not capture key social determinants of health, including housing conditions, caregiver education, and air quality, which are known to influence asthma outcomes, limiting the scope of contextual interpretation.

## Conclusions

Our study has revealed that asthma remains a major contributor to pediatric ED utilization, with no notable decline over the 15-year period, indicating persistent gaps in preventive care and disease control. An association was also observed between socioeconomic status and asthma-related ED visits. Children with Medicaid were far more likely to present with asthma than those with private insurance, highlighting disparities in access to outpatient management. Additionally, racial and ethnic disparities were evident: Non-Hispanic Black children and those categorized as Non-Hispanic “Other” had significantly higher odds of asthma-related ED visits compared to their Non-Hispanic White peers, even after adjusting for demographic and clinical factors. This points to entrenched inequities in environmental exposures, healthcare access, and chronic disease outcomes. Gender differences were also notable, with male children accounting for a greater share of asthma-related ED visits, an observation that contrasts with traditional patterns in pediatric asthma. Ambulance use was low and consistent across groups, suggesting that most cases were not perceived as severe emergencies but still reflect gaps in routine asthma control. As noted earlier, asthma has remained a persistent driver of pediatric ED visits over the past 15 years, underscoring unmet needs in preventive care and the stark inequalities associated with Medicaid coverage, gender, and race. Addressing these gaps requires targeted solutions, including Medicaid reforms to improve access to inhalers and specialists, effective community health worker programs for high-risk groups, and updated clinical guidelines with equity-focused protocols. Multilevel interventions, ranging from environmental policies to school-based asthma management, are also vital to reducing preventable ED utilization and mitigating systemic inequities in care.
